# Embryonic Fibroblasts Promote Antitumor Cytotoxic Effects of CD8^+^ T Cells

**DOI:** 10.3389/fimmu.2018.00685

**Published:** 2018-04-13

**Authors:** Yingyu Qin, Jung Hoon Shin, Jeong-Ho Yoon, Se-Ho Park

**Affiliations:** ^1^Department of Life Sciences, Korea University, Seoul, South Korea; ^2^ImmunoMax Co., Ltd, Korea University, Seoul, South Korea

**Keywords:** mouse embryonic fibroblast-conditioned medium, CD8^+^ T cells, cytotoxic T lymphocytes, long-term persistence, adoptive T cell therapy

## Abstract

Adoptive CD8^+^ T cell therapy has emerged as an important modality for the treatment of cancers. However, the significant drawback of transfused T cells is their poor survival and functionality in response to tumors. To overcome this limitation, an important consideration is exploring a culture condition to generate superior antitumor cytotoxic T lymphocytes (CTLs) for adoptive therapy. Here, we provide a novel approach to generate potent CTL clones in mouse embryonic fibroblast-conditioned medium (MEF-CM). We found CTLs derived with MEF-CM have higher potential in long-term persistence in tumor bearing and non-tumor-bearing mice. Importantly, adoptive transfer of MEF-CM-cultured CTLs dramatically regressed tumor growth and prolonged mice survival. Characterization of MEF-CM-cultured CTLs (effector molecules, phenotypes, and transcription factors) suggests that MEF-CM enhances the effector functions of CD8^+^ T cells in part by the upregulation of the T-box transcription factor eomesodermin. Consequently, MEF-CM enhances the intrinsic qualities of effector CD8^+^ T cells to augment antitumor immunity.

## Introduction

The transfusion of T lymphocytes, referred to as adoptive T cell therapy, presents a promising approach to treat patients with cancers or infections (1, 2). Early works show that CD8^+^ T cells are an optimal lymphocyte population for adoptive transfer (3, 4). Especially in recent years, a growing number of engineered T cells with desired antigen specificity have been generated and expanded to overcome the limitation of low frequency of tumor-specific T cells. Examples are T cells engineered to express chimeric antigen receptors or engineered T cell receptors (5). Nevertheless, treatment of *in vitro* expanded CD8^+^ T cells does not consistently translate into an objective clinical tumor response. This suggests that *in vitro*-generated T cells are less effective in tumor killing or the effector cells have a short life span after infusion by activation-induced cell death (6–10). To overcome the limitations, an important consideration is generation of superior antitumor cytotoxic T lymphocytes (CTLs) by optimizing the *in vitro* culture conditions (7, 11–13).

The plastic culture vessels currently used to expand T cells *in vitro*, however, hardly replicate the *in vivo* environment. Alternatively, a desirable feeder cells could provide T cells a direct contact to mimic *in vivo* environment. Fibroblasts comprise heterogeneous tissue connecting cells that extensively distribute in organs of animals and play a critical role in wound healing through production of extracellular matrix (ECM), matrix metalloproteinase, and cytokine mediators (14, 15). There is evidence that ECM produced by fibroblasts serves as co-stimuli to enhance T cells activation and proliferation (16, 17). In addition, fibroblasts produce many molecules with the potential to modulate T cells functions. For example, fibroblasts derived from human lung tumors or normal skin can improve the production of interferon-gamma (IFN-γ) and interleukin (IL)-17A by T cells through secretion of soluble factor(s) (18). Another concept is that fibroblasts derived factor(s) also enhance the survival of activated T cells (19). The comprehensive effects of fibroblasts on T cells may potentially allow the alteration of the fate or intrinsic functions of T cells, which could be utilized in an *in vitro* culture system for adoptive cell therapy.

Mouse embryonic fibroblasts (MEFs) are stem cell-like fibroblasts that are widely used as feeder cells, since they secret various growth factors to support embryonic stem cells self-renewal and growth in an undifferentiated state. We were therefore interested in exploring whether MEFs are desirable candidates for facilitating the differentiation of potent effector CTL clones for adoptive cell therapy. Surprisingly, we found that MEFs enhanced effector functions of CD8^+^ T cells through soluble factor(s). Effector CD8^+^ T cells generated in mouse embryonic fibroblast-conditioned medium (MEF-CM) persisted long term after adoptive transfer. And in the murine tumor model, transfusion of short-term MEF-CM-cultured CTLs significantly regressed tumor growth.

## Materials and Methods

### Mice and Cells

Wild-type (WT) C57BL/6(B6) mice (Ly5.2^+/+^), BALB/c and ovalbumin (OVA)_257–264_-specific TCR (Vα2 and Vβ5) transgenic mice (OT-1) that were maintained on the B6 background were purchased from The Jackson Laboratory. Ly5.1^+/−^ OT-1 mice were obtained from OT-1 that were mated with B6 congenic mice Ly5.1^+/+^. All mice were 7–9 weeks old at the beginning of each experiment, and were raised in a specific pathogen-free environment at Korea University. The experimental protocols adopted in this study were approved by the Institutional Animal Care and Use committee of Korea University.

Primary MEFs were prepared from a pregnant B6 or BALB/c mice at 13 or 14 days post-coitum. MEFs after passage 2 (P2) were collected and maintained as stock cells. EG.7 tumor cells expressing chicken OVA were provided by Dr. M. Mescher (University of Minnesota, Minneapolis, MN, USA). MEFs were maintained in Dulbecco’s modified Eagle’s medium (DMEM, Gibco) supplemented with 10% fetal bovine serum (FBS), 2 mM l-glutamine, 1% penicillin-streptomycin, 10 µg/mL gentamycin, and 50 µM β-mercaptoethanol (Gibco-BRL). Primary MEFs (P3) from B6 or BALB/c were seeded with 1.25 × 10^5^/ml in DMEM supplemented with 10% FBS, 2 mM l-glutamine, 1% penicillin-streptomycin, 10 µg/mL gentamycin, and 50 µM β-mercaptoethanol (Gibco-BRL) and cultured for 2 days. The culture medium was collected by centrifuging for 5 min at 400 *g* followed by filtration through a 0.22-µm pore size filter and was stored at −85°C (conditioned medium, CM hereafter).

### *In Vitro* Activation of CD8^+^ T Cells

Splenic CD8^+^ T cells from OT-1 mouse were purified with a MACS column using anti-mCD8α magnetic beads (Miltenyl Biotec). The purity of the sorted OT-1 cells was >95%. Enriched OT-1 cells were stimulated with K^b^-OVA beads which consisted of OVA_257–264_ (Genscript) loaded recombinant MHC class I molecules (H2-K^b^) and anti-CD28 antibodies coated on magnetic beads. For the preparation of MHC-I beads, 1 µg of biotinylated H2-K^b^-OVA_257–264_, 0.3 µg of biotinylated anti-CD28 antibodies and 0.05 µg of streptavidin magnetic beads [NEB, S1420S] were incubated for overnight at 4°C with rotation. During cell stimulation, OT-1 cells were co-cultured with or without MEF feeder cells, which were seeded before CTL activation. Otherwise, OT-1 cells were cultured in the presence or absence of CM instead of MEF cells.

For assessment of CD8^+^ T cell proliferation, purified 1 × 10^5^ OT-1 CD8^+^ T cells were labeled with 2.5 µM carboxyfluorescein diacetate succinimidyl ester (CFSE) and activated by K^b^-OVA beads. After 2 days of activation, proliferation was assessed by CFSE dilution using fluorescence-activated cell sorting (FACS) analysis.

### Trans-Well Assay

Trans-wells (6.5 mm) with 0.4-µm pores were utilized (Corning). OT-1 CD8^+^ T cells (1 × 10^5^) alone or together with 2.5 × 10^5^ B6 MEFs in 100 µl RPMI-1640 medium were added to the upper chamber and 600 µl of medium with or without 2.5 × 10^5^ MEFs were added to the lower chamber. After 3 days of stimulation with K^b^-OVA beads, IFN-γ from the supernatant in the lower chamber was measured by ELISA, and IFN-γ-producing cells were detected by flow cytometry after intracellular staining.

### Flow Cytometry

The following antibodies were used for flow cytometry for cell surface and intracellular staining: TCRβ-fluorescein isothiocynate (FITC) (H57-597), CD25-FITC (3C7), Ly5.1-FITC (A20), IL-6-FITC (MQ2-13A5), TCRVα2-Phycoerithrin (PE) (B20.1), Ly5.2-PE (104), CD44-PE (IM7), granzyme B-PE (NGZB), Eomes-PE (Dan11mag), IL-1α-PE (ALF-161), IL-2-PE (MQ1-17H12), IL-3-PE (MP2-8F8), IL-4-PE (11B11), IL-13-PE (eBio13A), TNFα-PE (MP6-XT22), GM-CSF-PE (MP1-22E9), MCP-1-PE (2H5), Bcl-6-PE (K112-91), TCF-7/TCF-1-PE (S33-966), TCRVβ5.1,5.2-Allophycocyanin (APC) (MR9-4), CD62L-APC (MEL-14), IFN-γ-APC (XMG1.2), granzyme B-APC (NGZB), IL-17A-APC (ebio17B7), IL-5-APC (TRFK5), IL-10-APC (JES5-16E3), Perforin-APC (ebioOMAK-D), T-bet-APC (4B10), Blimp-1-Alexa Fluor^®^ 647 (5E7), CD8a-PerCP-Cyanine5.5 (53-6.7), B220-PerCP-Cyanine5.5 (RA3-6B2), streptavidin-APC.Cy7, and CD122-biotin (5H4). Flow cytometry was performed using a FACSVerse or FACSCalibur device (BD Biosciences) and data were analyzed using FlowJo_V10 (FlowJo LLC).

### *In Vitro* Cytotoxicity Assay

The ability of effector OT-1 CD8^+^ T cells to kill target cell EG.7 was evaluated by a CFSE/7-AAD-based flow cytometry assay as previously described (20). Briefly, effector cells (splenic CD8^+^ T cells) were sorted from OT-1 mice and were activated by K^b^-OVA beads for 3 days in the presence or absence of MEF-CM (50%, v/v) were labeled with CFSE. CFSE-labeled effector cells were incubated 4–5 h with target cells at an effector:target (E:T) ratio of 8:1, 4:1, 2:1, 1:1, and 0:1. Following a further wash, cells were stained with 7-AAD to identify dead cells. The cells were then analyzed *via* flow cytometer. Cytotoxicity was calculated on CFSE-negative cells as follows: %lysis = 100 × (% sample lysis − % basal lysis)/(100 − % basal lysis).

### Adoptive Transfer Studies

#### Cell Persistence Assay

For non-tumor-bearing mice, congenic Ly5.1^+/−^ effector CD8^+^ OT-1 cells (5 × 10^4^) generated in the presence or absence of B6 MEF-CM (50%, v/v) were intravenously transferred to Ly5.2^+/+^ B6 WT mice. After 1 month, transferred OT-1 cells were restimulated with of OVA_257–264_ peptides (100 μg/mouse) and the frequencies of OT-1 cells were measured in peripheral blood, spleen, and lymph node 5 days after peptide treatment. For tumor-bearing mice, OVA-expressing EG.7 tumor cells were transferred subcutaneously to WT B6 mice (1.5 × 10^5^ cells/mouse). When tumor size was 40–50 mm^3^ (10 days after tumor inoculation), medium cultured Ly5.1^+/+^effector CD8^+^ OT-1 cells (0.75 × 10^6^) and B6 MEF-CM (50%, v/v) cultured Ly5.1^+^Ly5.2^+^ effector CD8^+^ OT-1 cells were co-transferred to Ly5.2^+/+^ B6 WT mice. The persistence of transferred cells was analyzed after 7 and 14 days of T cell transfer, respectively.

#### Tumor Rejection Assay

For tumor rejection experiment, EG.7 tumor cells were transferred subcutaneously to WT B6 mice (5 × 10^4^ cells/mouse). When tumors were detectable (usually between 11 or 12 days after tumor inoculation), 1 × 10^6^ *in vitro*-generated effector cells were intravenously injected, and tumor sizes were measured every 2–4 days. Tumor sizes were calculated by determining the length of short (*l*) and long (*L*) diameters (tumor volume = *l*^2^ × *L*/2). Experimental endpoints were reached when tumor volume exceeded 2,500 mm^3^.

### Statistical Analysis

Prism software (GraphPad Prism6.0) was used for statistical analysis. Statistically significant differences were determined by unpaired *T*-test and one- or two-way ANOVA with Bonferroni’s multiple comparisons tests. Log-rank (Mantel–Cox) tests were used to analyze mouse survival curves. The bars in all graphs were expressed as mean ± SEM. In all figures, ns denotes no significance; *P < 0.05; **P < 0.01; ***P < 0.001; and ****P < 0.0001.

## Results

### MEFs Significantly Promote CD8^+^ T Cells Activation

**Figure 1:**
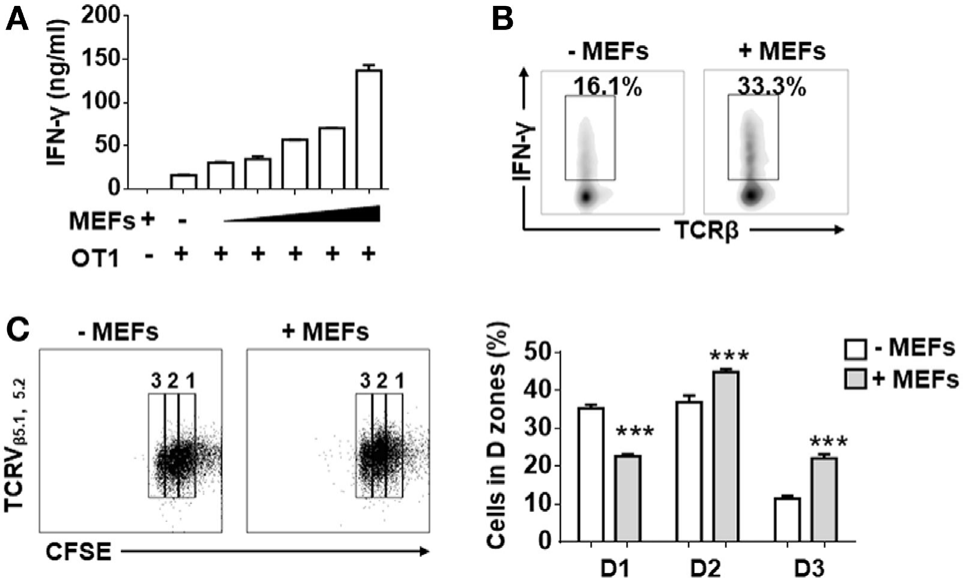
Mouse embryonic fibroblasts (MEFs) promote activation of CD8^+^ T cells activation. **(A)** Sorted CD8^+^ T cells from OT-1 splenocytes were activated by K^b^-OVA beads in the presence of different numbers of B6 MEF feeders [0, 0.3, 0.6, 1.3, 2.5, 5 × 10^4^/well (96 well, 200 µl culture medium)]. After 3 days of TCR stimulation, the level of interferon-gamma (IFN-γ) production was detected by ELISA. **(B)** On day 3, flow cytometry analysis of IFN-γ production in OT-1 CD8^+^ T cells in the presence or absence of B6 MEF feeders (2.5 × 10^4^/well) was determined. **(C)** Carboxyfluorescein diacetate succinimidyl ester (CFSE)-labeled OT-1 CD8^+^ T cells were stimulated in the presence or absence or B6 MEF feeders (2.5 × 10^4^/well), and after 2 days of TCR stimulation, the proliferative capacity of CD8^+^ T cells was determined by CFSE dilution. Statistical significance was analyzed by two-way ANOVA with Bonferroni’s multiple comparisons tests (***P ≤ 0.001). All data represent at least two independent experiments.

In order to determine whether MEFs influence the activities of antigen-recognized CD8^+^ T cells, isolated splenic CD8^+^ T cells from OT-1 mice were stimulated by K^b^-OVA beads in the presence of different numbers of autologous MEF feeders. Following 3 days of stimulation, IFN-γ production measured by ELISA positively correlated with the number of MEF feeders (Figure 1A). About a twofold enhancement of IFN-γ-producing cells for MEF-based cultures was confirmed by intracellular flow cytometry analysis (Figure 1B). Next, we evaluated the proliferative potential of CTLs in the presence of MEF feeders by the CFSE dilution assay. Following 48 h of stimulation, the CD8^+^ T cells in division zones 2 and 3 were increased in the presence of MEF feeders (Figure 1C), which indicated that MEF feeders also enhanced CTL proliferation.

### MEFs Enhance CD8^+^ T Cell Differentiation to Effector Cells Through Soluble Factor(s)

**Figure 2:**
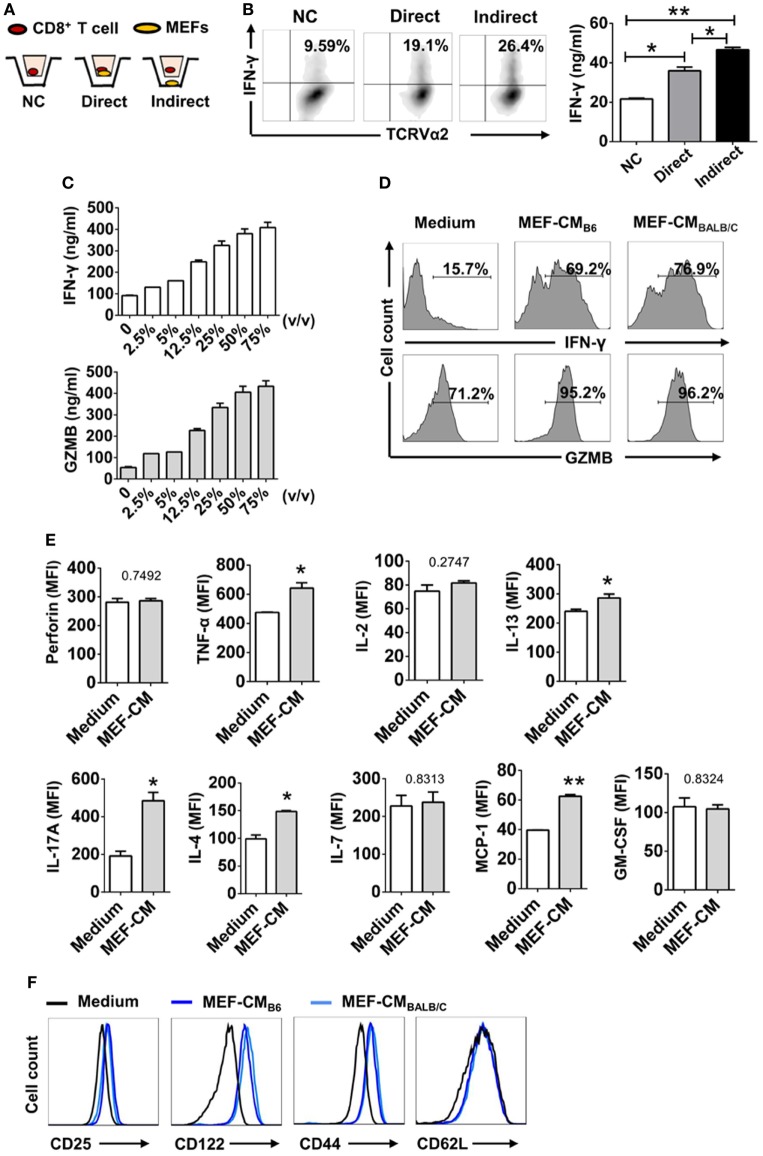
Mouse embryonic fibroblasts (MEFs) promote activation of CD8^+^ T cells through soluble factor(s). **(A)** Schematic diagram of trans-well co-culture system. **(B)** OT-1 CD8^+^ T cells (1 × 10^5^) were incubated alone (No MEFs) or together with 2.5 × 10^5^ B6 MEFs in the upper chamber (Direct), or CD8^+^ T cells in the upper chamber were incubated with 2.5 × 10^5^ MEFs in the lower chamber (Indirect). After 3 days of TCR stimulation, interferon-gamma (IFN-γ) production was evaluated by intracellular flow cytometry analysis. **(C)** OT-1 CD8^+^ T cells (1 × 10^5^) were stimulated with different volumes of B6 mouse embryonic fibroblast-conditioned medium (MEF-CM) for 3 days. IFN-γ and granzyme B (GZMB) levels were quantified by ELISA. **(D)** OT-1 CD8^+^ T cells were stimulated in the presence (50%, v/v) or absence of B6 MEF-CM or BALB/c MEF-CM. 2 days later, the level of IFN-γ and GZMB was determined by flow cytometry analysis. Statistical significance was analyzed by one-way ANOVA with Bonferroni’s multiple comparison test (*P ≤ 0.05; **P ≤ 0.01). **(E)** OT-1 CD8^+^ T cells were stimulated in the presence (50%, v/v) or absence of B6 MEF-CM for 2 days, the level of cytokines and chemokines was determined by flow cytometry analysis. Statistical significance was analyzed by unpaired *t*-test (*P ≤ 0.05; **P ≤ 0.01). **(F)** Surface markers were analyzed following 3 days stimulation by flow cytometry analysis. All data represent three independent experiments.

To determine whether MEFs could promote CTL activation through direct cell–cell contact or through soluble factor(s), the Trans-well co-culture system was utilized. After 3 days of TCR stimulation, IFN-γ production was enhanced in both direct and indirect contact (Figures 2A,B). In terms of IFN-γ production by OT-1 cells, indirect condition was not inferior to direct cell–cell contact condition. Therefore, it was very likely that MEFs promoted CD8^+^ T cell activation through soluble factor(s). In addition, to exclude the effect of direct cell–cell contact, CD8^+^ T cells were stimulated in different volumes of MEF-CM. Following 3 days stimulation, IFN-γ and granzyme B levels were proportional to the volume of treated MEF-CM (Figure 2C). In these experiments, we used MEF-CM produced from autologous C57/BL6 (B6, H-2^b^) MEF cells. To further analyze whether allogenic MEF-CM also has the same effects on CD8^+^ T cells, MEF-CM-mediated enhancement in OT-1 CD8^+^ T cells was compared between allogenic BALB/c (H-2^d^) MEF-CM and autologous B6 MEF-CM. Increased levels of IFN-γ and granzyme B were also observed in BALB/c MEF-CM treatment, which was similar with the effects of B6 MEF-CM (Figure 2D). Cytokine expression profile between conventional medium and MEF-CM-cultured CTLs was further compared. We observed MEF-CM significantly elevated IL-17A and also slightly elevated TNF-α, IL-4, IL-13, and GM-CSF expression. However, there were no significant differences in the expression of perforin, IL-7, and MCP-1 between the two cultures (Figure 2E; Figure S1 in Supplementary Material). IL-5, IL-6, and IL-10 were undetectable in both of the two cultures (data not shown). Collectively, these results indicated that MEF-CM promoted the enhanced production of some effector molecules by CTLs following TCR engagement.

Since typical surface markers are another hallmarks for effector cell differentiation, surface markers of CTLs were analyzed following 3 days of TCR stimulation in the presence or absence of MEF-CM (50%, v/v). Effector markers CD25 (IL2Rα) and CD122 (IL2Rβ), responsible for IL-2-mediated proliferation, were dramatically upregulated in the presence of either B6 or BALB/c MEF-CM (Figure 2E). Another effector marker, CD44, a glycoprotein involved in cell adhesion and migration, was also significantly induced by MEF-CM. With respect to naïve marker CD62L, there was no significant difference between medium cultured and MEF-CM-cultured CTLs (Figure 2F). Taken together, these results support the idea that MEF-CM augments antigen-induced differentiation of CD8^+^ T cells into effector CD8^+^ T cells, and may be capable of enhancing the intrinsic functions of effector CD8^+^ T cells.

### MEF-CM Strongly Induces Expression of Eomesodermin

**Figure 3:**
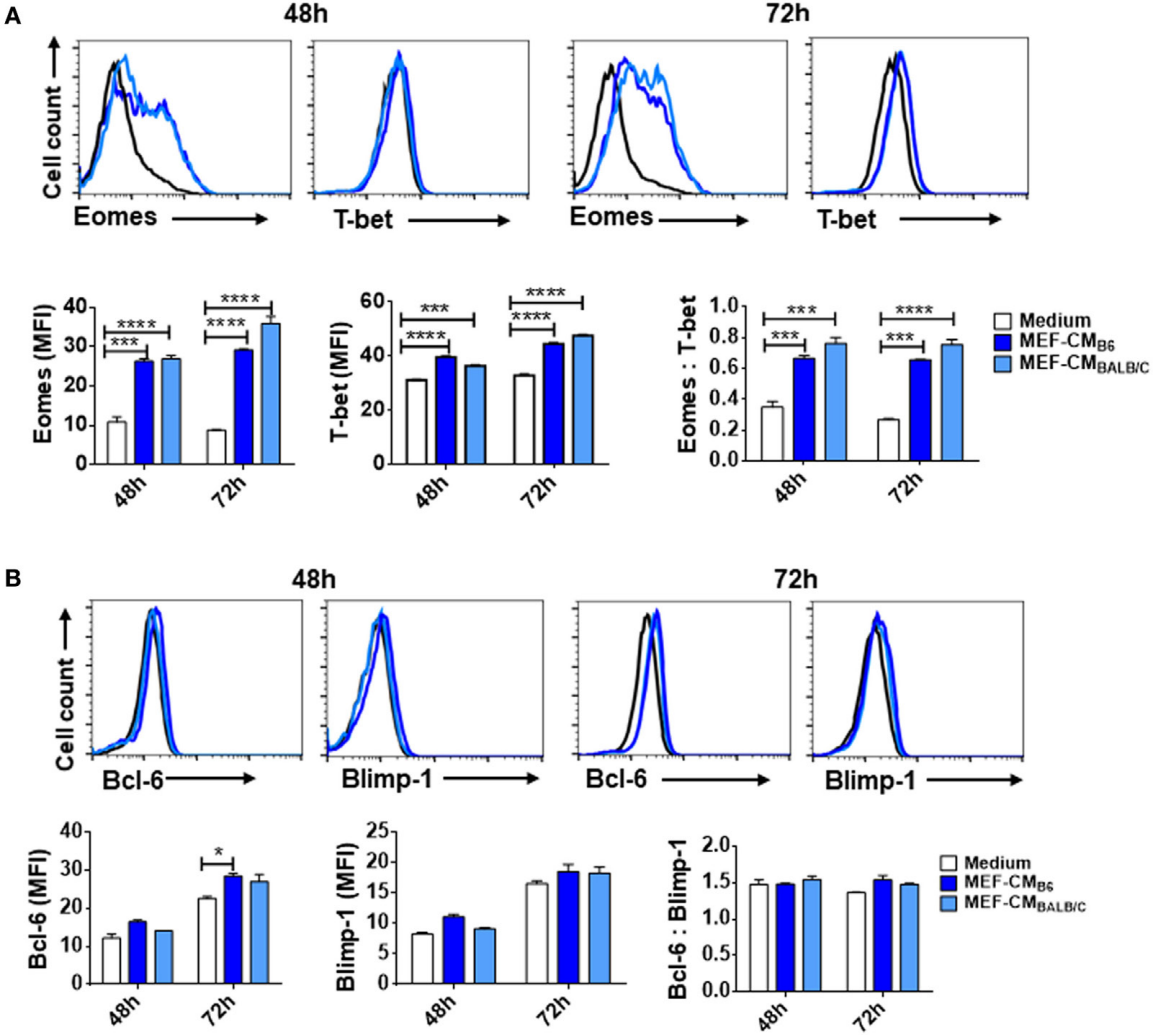
Mouse embryonic fibroblast-conditioned medium (MEF-CM) strongly induces eomesodermin expression. OT-1 CD8^+^ T cells were stimulated in the presence or absence of MEF-CM (50%, v/v). **(A,B)** T-bet, Eomes, Bcl-6, and Blimp-1 were examined after 48 and 72 h stimulation using intracellular fluorescence-activated cell sorting analysis. Histograms represented the mean fluorescence intensity (MFI) values of the transcription factors (left and middle), and the ratio of MFI for Eomes relative to T-bet or Bcl-6 to Blimp-1 (right). Data represent at least three independent experiments with similar results. Statistical significance was analyzed by two-way ANOVA with Bonferroni’s multiple comparison test (*P ≤ 0.05; ***P ≤ 0.001; ****P ≤ 0.0001).

The T-box transcription factors T-box expressed in T cells (T-bet) and Eomesodermin (Eomes) have been implicated as master regulators of CD8^+^ T cell differentiation and function (21, 22). To assess the effects of MEF-CM on transcriptional regulation of effector CD8^+^ T cell differentiation, intracellular levels of T-bet and Eomes of CD8^+^ T cells were measured in the presence or absence of MEF-CM following antigen priming. As shown in Figure 3A, Eomes was significantly elevated only in MEF-CM-cultured CTLs, whereas Eomes was always expressed in a low level in the absence of MEF-CM during 72 h stimulation (Figure 3A, left panel). With respect to T-bet, both of medium cultured and MEF-CM-cultured CTLs expressed high level of T-bet (Figure 3A, middle panel). Consequently, the ratio of Eomes:T-bet, which is critical for the regulation of memory cell differentiation, was dramatically increased in MEF-CM culture conditions (Figure 3A, right panel). In addition, another antagonistic transcription factors Bcl-6 and Blimp-1 that also control effector and memory cell differentiation were also examined (Figure 3B). Although a modest increase of Bcl-6 was observed in MEF-CM treatment, there was no significant difference in the expression of Blimp-1 as well as the ratio of Bcl-6:Blimp-1 that is critical for conversion to memory cells (23, 24). Given the notion that T-bet and Eomes cooperatively or alternatively promote cytotoxic lymphocyte formation by inducing the expression of IFN-γ and cytolytic molecules (21, 22), these data suggest that MEF-CM-mediated upregulation of IFN-γ and granzyme B could be a result of strongly induced Eomes.

### Antibody Array Analysis of Cytokines Secretion From MEFs

The cytokine environment is a key factor governing the direction of T cell differentiation (25–27). For example, IL-2 and IL-4 are capable of inducing Eomes expression to modulate the fate and function of cells (12, 28, 29). In order to investigate whether cytokines secreted by MEFs were responsible for CD8^+^ T cell modulation, MEF-CM (B6 and BALB/c) cytokine arrays were used. We could not observe cytokines that are known to modulate T-cell functions such as IL-2, IL-4, and IL-12 on the array (Figure S2 in Supplementary Material). The result indicates that at least IL-2 and IL-4 were not the major factors for the MEF-CM-mediated enhancement of T cell activation. Rather, there may be another factor(s) produced by MEF that improves CTLs effector functions. Furthermore, a recent report proposed that MEF feeders enhance hESCs differentiation to cardiomyocytes through the co-activation of Wnt3 and Eomes (30). It was also validated that induction of Wnt3-β-catenin signaling promotes memory CD8^+^ T cell differentiation (31, 32). To know whether there were Wnt ligands in MEF-CM to modulate T cell functions, we examined whether MEF-CM enhanced the level of TCF-7/TCF-1 (transcription factor-7, also known as T cell factor 1) that is downstream of Wnt-β-catenin pathway. We observed MEF-CM did not influence CD8^+^ T cells in the expression of TCF-7/TCF-1 (Figure S3 in Supplementary Material). Therefore, the MEF-CM effects we observed may not dependent on Wnt-β-catenin signals.

### MEF-CM Enhances CTLs Cytotoxicity

**Figure 4:**
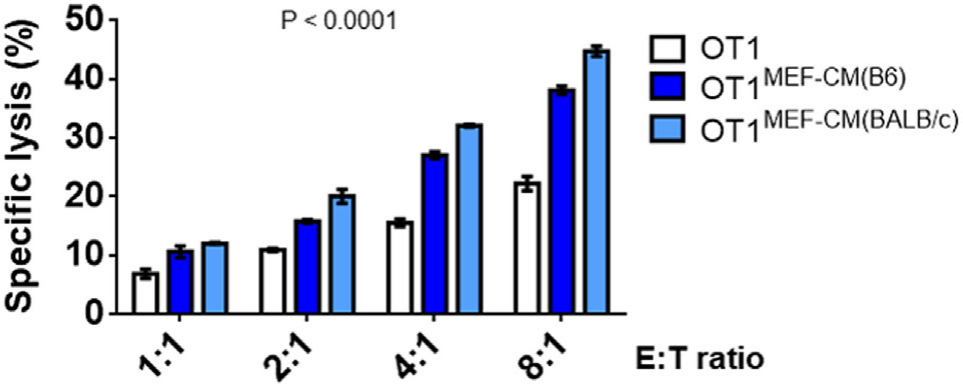
Specific lysis of EG.7 targets by effector OT-1 cytotoxic T lymphocytes (CTLs), effector OT-1 CD8^+^ T cells were prepared by the stimulation for 3 days in the presence or absence of mouse embryonic fibroblast-conditioned medium (MEF-CM) (50%, v/v). EG.7 cells which expression OVA was used as target cells. Effector cells and target cells were incubated 4–5 h in E:T ratios ranging from 1:1 to 8:1. To prevent the contamination of effector cells from the analysis of target cell lysis OT-1 CTLs were labeled with carboxyfluorescein diacetate succinimidyl ester and excluded in fluorescence-activated cell sorting analysis. The cell death was measured by 7-AAD incorporation. Data represent at least three independent experiments with similar results. Statistical significance was analyzed by two-way ANOVA with Bonferroni’s multiple comparison test.

As elevated IFN-γ and granzyme B production by CTLs in the presence of MEF-CM was observed (Figure 2), we further examined the ability of CTL-mediated lysis to target tumors *in vitro*. The flow cytometry-based cytotoxicity assay was performed using EG.7 OVA-expressing tumor cells as targets. As expected, the cytotoxic capacity of CTLs derived with MEF-CM was much higher than that of conventional medium cultured CTLs (Figure 4). Collectively, the data concerning the production of effector molecules, expression of phenotypic cell surface makers, and *in vitro* cytotoxicities indicate that MEF-CM has potential to modulate CD8^+^ T cell activation toward more potent CTLs.

### CTLs Derived With MEF-CM Are Very Likely to Survive After Adoptive Transfer

**Figure 5:**
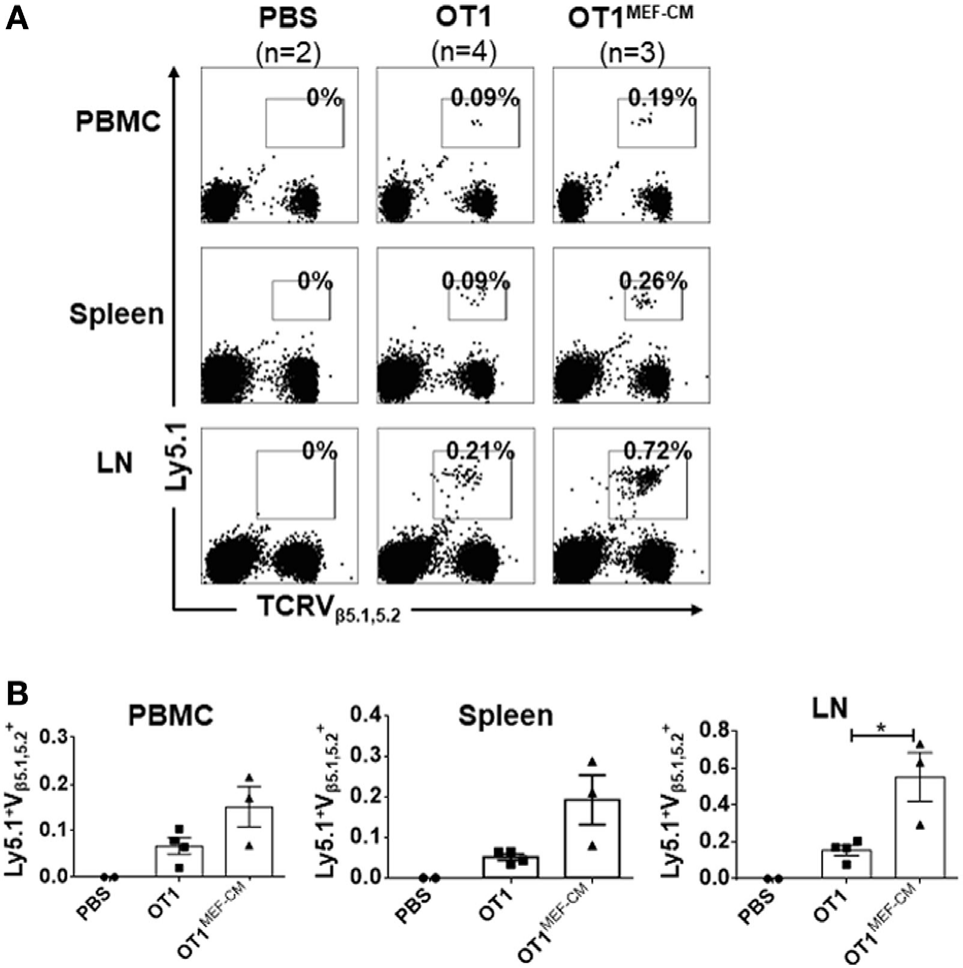
High potential of mouse embryonic fibroblast-conditioned medium (MEF-CM) cultured cytotoxic T lymphocytes in long-term persistence and proliferation after adoptive transfer. **(A,B)** Ly5.1^+/−^ OT-1 cells (5 × 10^4^) that were generated in the presence or absence of MEF-CM (50%, v/v) were transferred to Ly5.2^+/+^ B6 wild-type mice. After 1 month, mice were subcutaneously administrated with OVA_257–264_ peptide antigen and the number of Ly5.1^+/−^ OT-1 cells were determined from blood, spleen, and lymph node 5 days later. **(A)** A representative fluorescence-activated cell sorting result. **(B)** Summary of result **(A)** with recipient groups transferred with PBS alone (two mice), OT-1 cells (four mice), and OT-1 cells cultured with MEF-CM (three mice). Data are representative of two independent replicates. Statistical significance was analyzed by two-way ANOVA with Bonferroni’s multiple comparison test (*P ≤ 0.05).

**Figure 6:**
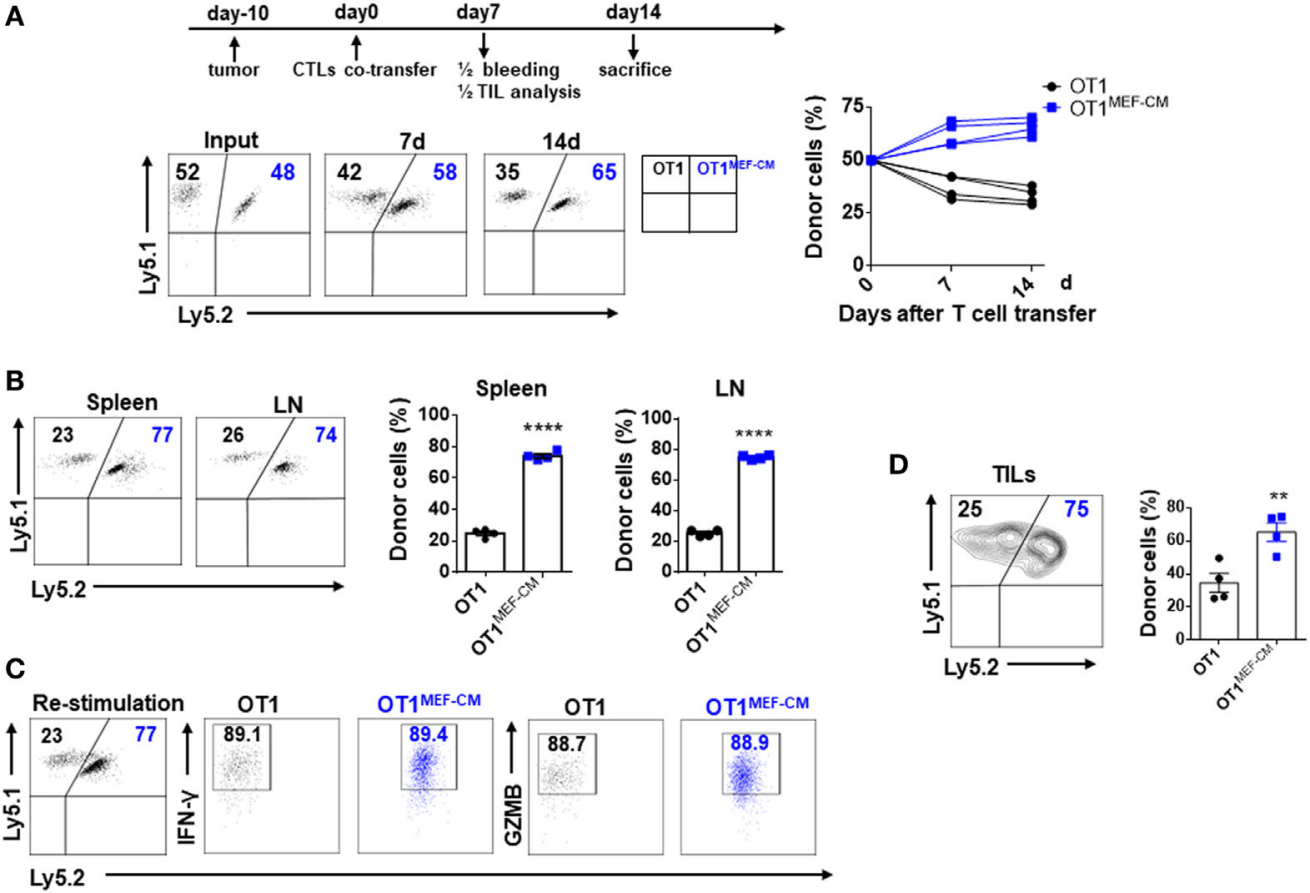
Mouse embryonic fibroblast-conditioned medium (MEF-CM)-educated cytotoxic T lymphocytes (CTLs) exert higher potential of persistence in tumor-bearing mice. Medium cultured Ly5.1^+/+^ OT-1 (0.75 × 10^6^) and MEF-CM culture Ly5.1^+^Ly5.2^+^ OT-1 (0.75 × 10^6^) were co-transferred to tumor bearing (40–50 mm^3^) Ly5.2^+/+^ B6 mice (*n* = 8). The frequency of OT-1 cells were determined in peripheral blood **(A)** on days 7 and 14 of T cell transfer, and spleen and lymph node **(B)** on day 14 of T cell transfer. A representative fluorescence-activated cell sorting result showing percentages in plots indicate percent of donor cells among Ly5.1^+^ CD8^+^ T cells. Graphs are the summary of the dot plots on the left with each dot representing for a recipient (*n* = 4). **(C)** Whole splenocytes were restimulated with OVA_257–264_ peptides for 2 days *in vitro*, interferon-gamma, and granzyme B-producing levels were examined. **(D)** Transferred cells in the tumors were analyzed after 7 days of T cell transfer. Half of the recipients (*n* = 4) were analyzed for panels **(A–C)** and the remaining were analyzed for panel **(D)**. Statistical significance was analyzed by unpaired *t*-test (**P ≤ 0.01; ****P ≤ 0.0001). Two independent biological experiments were proceeded with similar results.

Mouse embryonic fibroblast-derived factor(s) strongly induced transcription factor Eomes (Figure 3A), which is considered necessary for memory CD8^+^ T cell differentiation (33–35). Therefore, we hypothesized that MEF-CM-cultured CTLs may acquire the potential for long-term survival after primary activation. To validate this assumption, *in vitro*-generated effector Ly5.1^+/−^ OT-1 in the presence or absence of MEF-CM were adoptively transferred to Ly5.2^+/+^ B6 mice. One month later, the sustained population of transferred cells in tissues (peripheral blood, spleen, and lymph node) was analyzed. The frequency of Ly5.1^+/−^ OT-1 cells was significantly increased in the group transferred with MEF-CM-educated OT-1 cells (Figures 5A,B). To further analyze whether MEF-CM improves CTLs survival in tumor-bearing mice, medium cultured Ly5.1^+/+^ OT-1 and MEF-CM culture Ly5.1^+^Ly5.2^+^ OT-1 with ratio of 1:1 were co-transferred to tumor bearing Ly5.2^+/+^ B6 mice. After 11 days T cell transfer, tumor was totally rejected. The persistence of donor cells was examined in peripheral blood on day 7 and day 14, respectively. As expected, the frequency of MEF-CM-cultured CTLs was gradually increased, whereas medium cultured CTLs was decreased (Figure 6A). The persisted cells in other tissues (spleen and lymph node) were also compared on day 14. Notably, higher frequency of MEF-CM-cultured CTLs was homing to secondary lymphoid organs comparing to that of medium cultured CTLs (Figure 6B). The effector function of the transferred cells was compared by *in vitro* re-stimulation. The producing level of IFN-γ and granzyme B was comparable between MEF-CM and conventional medium cultured CTLs (Figure 6C). To analyze the ability of transferred cells in tumor infiltrating, tumor-infiltrating cells were examined on day 7, consistence with the results of other tissue migration, higher frequency of MEF-CM-cultured CTLs infiltrated to tumors (Figure 6D).

### CTLs Derived With MEF-CM Effectively Regress Tumors

**Figure 7:**
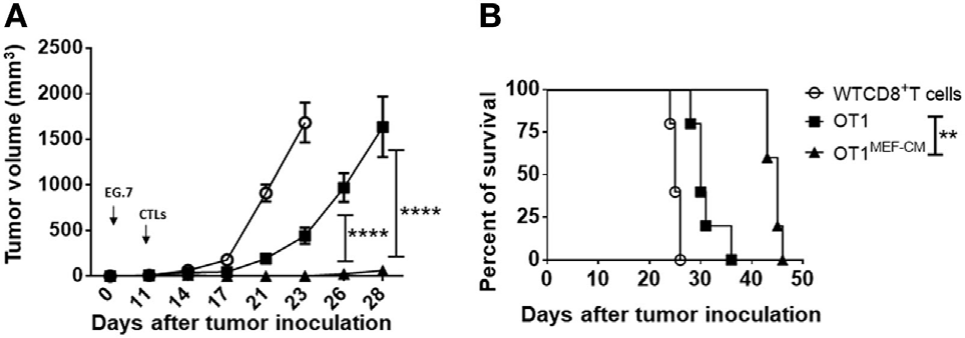
Potent *in vivo* antitumor immunity of mouse embryonic fibroblast-conditioned medium (MEF-CM)-educated cytotoxic T lymphocytes (CTLs). **(A,B)** EG.7 tumor cells were subcutaneously inoculated in B6 WT mice. After 10–11 days, tumors were detectable. Adoptive transferred CD8^+^ T cells which were generated in the presence or absence of MEF-CM (50%, v/v). Tumor size and survival were monitored regularly. Serial tumor measurements were obtained. One representative experiment of three with *n* = 6 mice per group. Comparison of tumor size by two-way ANOVA with Bonferroni’s multiple comparison test. Comparison of survival curves with Log-rank (Mantel–Cox) test (**P ≤ 0.01; ****P ≤ 0.0001).

The ability of T cells to proliferate and survive for a long term after adoptive transfer is associated with effective antitumor immunity. Consequently, we investigated whether MEF-CM-educated CD8^+^ T cells were superior in the ability of tumor regression using solid EG.7 tumor model. *In vitro*-activated OT-1 cells generated in the presence or absence of MEF-CM (K^b^-OVA beads stimulation) were adoptively transferred into tumor bearing B6 WT mice. WT CD8^+^ T cells that lacked antigen specificity (anti-CD3/CD28 stimulation) were used as a negative control. Monitoring of tumor size demonstrated that MEF-CM-educated OT-1 CTLs had superior potential to regress the tumor growth compared with OT-1 cells cultured without MEF-CM (Figure 7A). In addition, improved survival was also observed in mice that received MEF-CM-educated OT-1 cells (Figure 7B).

## Discussion

Successful adoptive cell therapy for cancer is the outcome of multi-steps that depend on sufficient numbers of antigen-specific CTLs, survival and proliferation of CTLs after transfusion, and successful infiltration of CTLs to target sites. A deficiency at any step along this chain of events will reduce or even invalidate antitumor efficiency for infused CTLs (2, 3, 10, 36). Therefore, each step should be optimized to develop a curative antitumor therapy. Here, we provide a novel method to produce long-lived, functionally potent CTL clones using MEF-CM treatment cultures. Compared with CTLs cultured solely in medium, MEF-CM-cultured CTLs displayed greater potential for long-term survival and exerted superior antitumor immune response after adoptive transfer.

The original role of MEF cells in this study was as feeder cells to support CTLs *ex vivo* expansion, which provides CTLs a contact with “tissues.” Unexpectedly, the Trans-well experiment showed that Mouse embryonic fibroblasts dramatically enhanced the levels of IFN-γ and granzyme B of CTLs through the production of soluble factor(s) (Figures 2A–D). Hence, we further extended this investigation. We assessed that MEF-CM enhanced antigen-induced acquisition of cytolytic CD8^+^ T cell characteristics at the levels of phenotype expression (Figure 2F), transcriptional regulation (Figure 3), and cytolytic function (Figure 4). These results support the notion that fibroblasts have the capacity to modulate the function of T lymphocytes (18, 19, 37).

The activation of CD8^+^ T cells has largely been determined by expression of effector molecules, such as IFN-γ and granzyme B. Here, we observed these expression levels were dramatically enhanced in the presence of MEF-CM (Figure 2). T-bet and Eomes are key transcription factors in cytotoxic lymphocyte lineage differentiation through the regulation of IFN-γ, granzyme B, and perforin expression (21, 22, 35, 38). During 72-h stimulation, MEF-CM-cultured CTLs expressed high levels of Eomes, contrary to the medium alone cultured CTLs, which always displayed a low level (Figure 3A). Although T-bet was slightly enhanced in the presence of MEF-CM, it was true also in the culture without MEF-CM. In addition, Blimp-1 has also been reported to involved in granzyme B expression (39). However, there was no significant difference in the expression of Blimp-1 between MEF-CM- and medium cultured CTLs (Figure 3B). Collectively, it implies MEF-CM-mediated enhancement of IFN-γ and granzyme B is mainly influenced by the upregulation of Eomes. In addition, evidence supports the view that T-bet and Eomes are important for differentiation and maintenance of effector and central memory cells. T-bet directs effector cell differentiation, whereas Eomes is responsible for memory CD8^+^ T cells differentiation (33–35). Therefore, we hypothesized that strongly induced Eomes in MEF-CM-cultured CTLs enhances the survival of CTLs following *in vivo* transfer. To assess that, a low dose of congenic effector OT-1 CD8^+^ T cells generated in the presence or absence of MEF-CM were transferred to WT mice. MEF-CM-educated CD8^+^ T cells displayed more potential to differentiate to long-lived memory cells (Figures 5A,B). Very few CD8^+^ T cells cultured in the absence of MEF-CM with a low level of Eomes persisted after infusion. This result provides further support back up the viewpoint that effector CD8^+^ T cells with high level of Eomes lean to memory cell differentiation. In addition, co-adoptive transfer experiment in tumor-bearing mice further validated the superior persistence of MEF-CM-cultured CD8^+^ T cells (Figure 6). And as shown in Figure 7, MEF-CM-cultured CD8^+^ T cells significantly regressed tumor growth and prolonged mice survival following adoptive transfer. These results support the notion that T-box transcription factors, especially Eomes, are critical for CTL antitumor immunity (40, 41).

Mouse embryonic fibroblast was known to secrete diverse chemokines, cytokines, and growth factors. Forty cytokines arrays of MEF-CM revealed that the mainly detectable molecules were chemokines in both B6 and BALB/c (Figure S2 in Supplementary Material). Although cytokines such as IL-2 and IL-4 enhance Eomes expression in CD8^+^ T cells (12, 28, 29), we did not found any evidence of IL-2 and IL-4 expression through the antibody array. This result was consistent with an early investigation on MEF (42). It can be considered that Eomes upregulation is not due to IL-2 or IL-4. As a recent report proposed that MEF feeders enhance hESCs differentiation to cardiomyocytes through the co-activation of Wnt3 and Eomes (30), although MEFs work on different cells, this finding is partially consistent with our results in terms of Eomes induction.

In conclusion, we provide a novel method to convert the fate of differentiated T cells from exhaustion to vigor, which overcomes the need for repetitive infusion of high dose of CD8^+^ T cells due to most of *in vitro* expanded CTLs loss of survival and functions. To our knowledge, this is the first study to investigate whether embryonic fibroblasts have the ability to enhance the intrinsic qualities of effector CD8^+^ T cells with promising outcomes in adoptive cancer therapy. Here, we achieved strong antitumor activity and prolonged survival of antigen-specific CTLs in just 3 days of culture. Our findings provide a clue to overcome the limitation of T cell based adoptive therapy, such as inferior cytotoxicity and a short life span, of *in vitro*-cultured CTLs. Identifying molecular factor(s) of MEF-CM responsible for the CTL potentiation will further shed light to anti-cancer T cell therapy.

## Ethics Statement

The experimental protocols adopted in this study were approved by the Institutional Animal Care and Use committee of Korea University.

## Author Contributions

YQ and S-HP designed the experiments; YQ performed the experiments; YQ and S-HP undertook the analysis of data. JS and J-HY reviewed data and manuscript preparation. YQ and S-HP wrote the paper.

## Conflict of Interest Statement

The authors declare that the research was conducted in the absence of any commercial or financial relationships that could be construed as a potential conflict of interest.
