# Supplementary material for: Embryonic Fibroblasts Promote Antitumor Cytotoxic Effects of CD8+ T Cells
Source: Front Immunol. 2018 Apr 13;9:685. doi: 10.3389/fimmu.2018.00685 (PMC5908885; doi:10.3389/fimmu.2018.00685)
Supplement: Supplementary file 1 [file Presentation_1.PDF]

Supplementary Material

Embryonic Fibroblasts Promote Antitumor Cytotoxic Effect of CD8$^+$ T Cells

Yingyu Qin*, Jung Hoon Shin, Jeong−Ho Yoon

*** Correspondence:** Se-Ho Park : sehopark@korea.ac.kr

Supplementary Figures

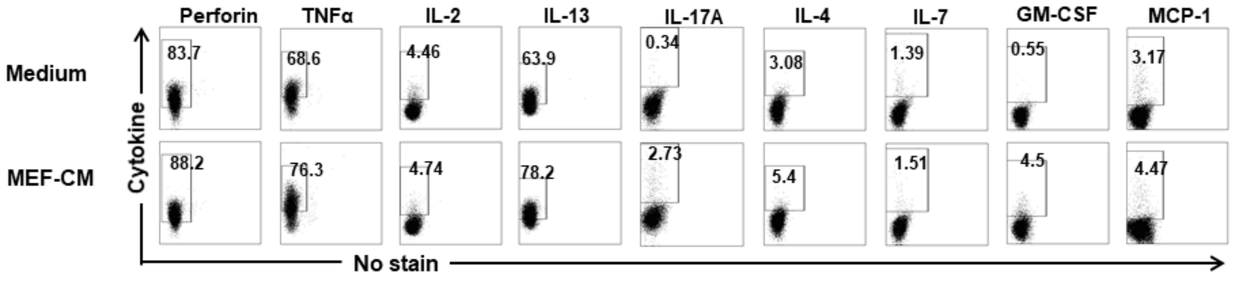

Supplementary Figure 1. **Expression profile of cytokines and chemokines determined by intracellular FACS analysis.**

OT-1 CD8$^+$ T cells were stimulated in the presence (50%, v/v) or absence of B6 MEF-CM for 2 days, the level of cytokines and chemokines was determined by flow cytometry analysis.

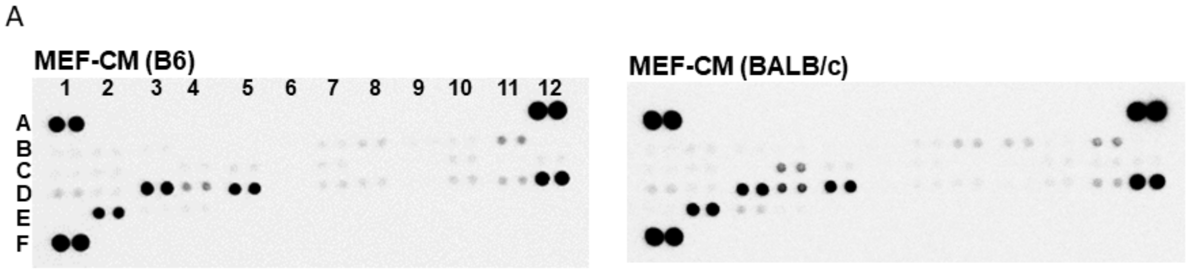

B

Positions of anti-cytokine antibodies on the array

	1	2	3	4	5	6	7	8	9	10	11	12
A	PC											PC
B	CXCL13	C5/C5a	G-CSF	GM-CSF	CCL1	CCL11	sICAM-1	IFN-γ	IL-1α	IL-1β	IL1Rα	IL2
C	IL-3	IL-4	IL-5	IL-6	IL-7	IL-10	IL-13	IL12p70	IL-16	IL-17	IL-23	IL-27
D	IP-10	CXCL11	CXCL1	M-CSF	MCP-1	CCL12	CXCL9	CCL3	CCL4	CXCL2	RANTES	CXCL12
E	CCL17	TIMP-1	TNFα	TREM-1								
F	PC											NC

Supplementary Figure 2. Cytokine expression profile determined by antibody array.

MEF-CM was prepared as previous shown. The array membrane with 40 different mouse cytokine antibodies was from R&D systems. Assays were performed according to the manufacturer's instructions A. Secreted proteins in B6 and BALB/c MEF-CM were detected using an array membrane with 40 kinds of cytokines and chemokines. B. Positions of anti-cytokine antibodies on the array. The grey background with white lettering represented the non-detectable cytokines that shared between B6 MEF-CM and BALB/c MEF-CM.

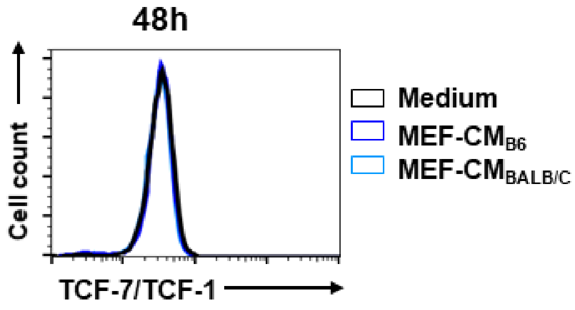

Supplementary Figure 3. MEF-CM dose not influence Wnt-β-catenin signaling in CTLs

OT-1 $CD8^+$ T cells were stimulated in the presence (50%, v/v) or absence of B6 or BALB/c MEF-CM, level of TCF-7/TCF-1 was examined at indicated time point by intracellular flow cytometry. A representative histogram displayed on 48h stimulation was presented.
